# AN EXPLORATION OF CULTURAL INFLUENCES ON SUBJECTIVE SUCCESSFUL AGING

**DOI:** 10.1093/geroni/igac059.1059

**Published:** 2022-12-20

**Authors:** Steffi Kim

**Affiliations:** University of Minnesota, Minneapolis, Minnesota, United States

## Abstract

Indigenous peoples worldwide face unique challenges growing old. Many of these challenges are remnants of previous colonization practices and current oppressive systems often leading to out-migration from rural to urban environments. Despite Anchorage having the highest population of Alaska Native Elders little is known about the experience of relocation. This study investigated the impact of culture on the experience of successful aging within the Alaska Native context. Twenty-five semi-structured qualitative interviews with rural (N=13) and urban Elders (N=12; ages 48-84) were conducted. The use of Gee’s discourse analysis tools provided the framework for analyzing the discourse of Elders based on location and traditional or western influences on subjective successful aging. We explored the use of language within two identified discursive patterns: cultural discourse and Elder identity discourse. Social and contextual determinants of successful aging involve aspects of minority and majority culture and self-appraisal of successful aging based on cultural assumptions.

